# Method for microplastics extraction from Lake sediments

**DOI:** 10.1016/j.mex.2020.101140

**Published:** 2020-11-12

**Authors:** M. Zobkov, M. Zobkova, N. Galakhina, T. Efremova

**Affiliations:** Northern Water Problems Institute of Karelian Research Centre of the Russian Academy of Sciences 50, Alexander Nevsky prospekt, Petrozavodsk 185030, Republic of Karelia, Russia

**Keywords:** Microplastics;Freshwater;Sediments;Identification;Potassium formate

## Abstract

In recent years, microplastics (MPs) contamination has been emphasized as one of the most global ecological problems. Bottom sediments were identified as one of the major sinks of MPs, while river-lake systems - as the main transport routs of MPs into the coastal seas and the World Ocean. Thus, the MPs determination in freshwater sediments is an important task for identifying sedimentation processes and contamination sources. Density separation is commonly used to extract anthropogenic particles from bottom sediments. The proposed method is dedicated to determining the abundance of MPs in freshwater organic-rich sediments. Repeated wet peroxide oxidation was applied to digest organic matter that can hamper the microplastic determination in a sample. Potassium formate with a density 1.5 g/mL was used as a separation solution. This method is intended to identify various types of MPs (fibers, films, fragments and beads) ranging in size from 0.174 to 5 mm. The effectiveness of the MPs extraction with potassium formate was 98.0 ± 2 % (*р* = 0.05; *n* = 16), while the extraction of MPs when analyzed under a microscope was 92.0 ± 4% (*р* = 0.05; *n* = 20).

## Highlights

• A new method for microplastic extraction from lake sediments is proposed.
• Potassium formate was applied for density separation.
• Repeated wet peroxide oxidation was applied to digest organic matter.
• Quality control procedures are proposed.
• The effectiveness of the microplastic extraction with potassium formate is 98.0 ± 2%.

## Graphical abstract

**Figure:**
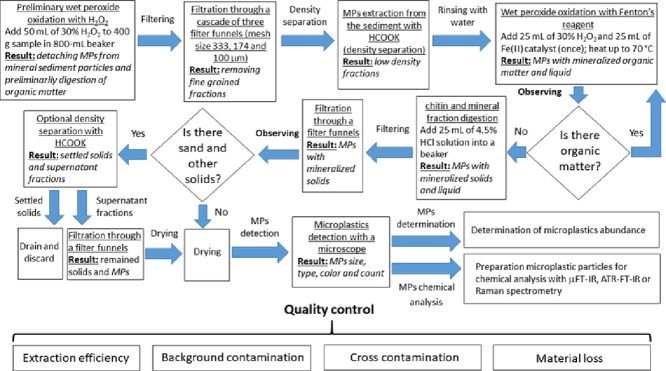

**Specifications Table**

**Table:** 

Subject Area	Environmental Science
More specific subject area	Assessment of microplastics abundance in lake sediments
Protocol name	Method for microplastics extraction from lake sediments
Reagents/tools	The method did not require no any specific equipment or resources and can be reproduced in any chemical laboratory. Glassware: Petrie dishes, glass beakers, water bath, glass funnels, desiccator, squirt bottles, filter nets with mesh size 333, 174 and 100 mm, stereomicroscope Reagents H_2_O_2_, HCl, HCOOK, FeSO_4_·7H_2_O, distilled water
Experimental design	Method involves preliminary wet peroxide oxidization of sediment sample, sediment flushing to remove fine grained fractions, density separation with potassium formate, wet peroxide oxidization, HCl digestion, optional density separation, filtration, drying and microplastics analysis under the microscope.
Trial registration	*n/a*
Ethics	*n/a*
Value of the Protocol	*Describe the importance of the protocol in up to 3 bullet points* • *Allows microplastics extraction from organic-rich sediments* • *Quality assurance and control procedures are provided*

## Method background

Despite the fact that microplastics (MPs) contamination represents a major global ecological problem, there is still no standardized method for extracting and quantifying MPs from environmental matrices. Density separation is commonly applied to isolate MPs from bottom sediments [1]. This technique is based on extraction of relatively light anthropogenic particles from heavier sediment grains using a separation solution [2]. Different concentrated salt solutions, such as NaCl, ZnCl_2_, NaI, CaCl_2,_ and others, are most commonly used to isolate MPs.

The proposed method is based on the NOAA laboratory methods for MPs analysis in the marine environment [7] with modifications [14,15]. As confirmed by Lenz et al [5] using Raman spectroscopy, the highest success rate of identification with a microscope was achieved for MPs larger than 100 µm. At the same time, as was demonstrated by Song et al [11], detection of some types of MPs < 200 µm was problematic using a microscope. Thus, we utilized MPs size near the 200 µm as the lower size limit for optimal MPs identification with a microscope.

MPs are polymers with a maximum dimension of less than 5 mm. Filters and nets are widely used to isolate MPs from other media. However, the size of the filtered items can be correlated with the mesh size of filters and meshes only for relatively spherical particles. At the same time, MPs have a wide range of forms: spheres, fibers, films and irregularly shaped fragments. As a result, in contrast to natural sediments which have a close to spherical form, the interaction of MPs with nets is more complicated. Elongated fibers and fragments can pass through a relatively small mesh size (much smaller than their maximum dimension). Films can also pass through the diagonal of a rectangular cell. This results in the loss of some MPs that have a minimum dimension smaller than the mesh diagonal. Thus, to retain particles of irregular shapes we used the mesh size of 174 µm as the main retention media, as they have a diagonal of 240 µm and the average value between the side and the diagonal of this mesh equals 207 µm. Therewith to retain elongated particles with a minimum dimension smaller than 174 µm, we also used a cascade of two filter funnels with filter nets installed one above the other.

In previous studies, it was found that a high content of organic matter in the sediment inhibits extraction of MPs [1,15]. Thus, the proposed method has been adapted to determine MPs in freshwater sediment that usually has a higher organic matter content than the same in marine sediments. For this purpose, the organic matter digestion was conducted in two steps. In the first step, preliminary wet peroxide oxidation with H_2_O_2_ was used to detach MPs from mineral sediment particles and partially digest the organic matter. MPs aggregation with sediment increases the bulk specific density of MPs which, in turn, can prevent plastics floatation during density separation. In the second step, wet peroxide oxidation with Fenton's reagent [12] was carried out after density separation to digest the remaining organic matter. To remove the fine fraction of sediment (mud and silt) that hinders separation of MPs, filtration through a cascade of three filter funnels (sediment flushing) was conducted before the density separation step.

At the stage of sediment flushing, large volumes of water are used to remove fine grained (mud and silt) sediment fractions. To reduce the loss of MPs during this procedure, a smaller filter net with a mesh size of 100 µm was installed at the bottom of the filter funnels cascade. Filtration using filter nets of 174 µm applied to subsequent stages removes smaller MP items retained in the flushing stage. A filter net with a mesh size of 333 µm was utilized in the flushing stage to reduce clogging of destination filter net with a mesh size of 174 µm.

Moreover, to enable further determination of heavy metals on extracted MP items, potassium formate with a density of 1.5 g/mL [13] was selected as the separation solution instead of ZnCl_2_, which was used in previous studies [14,15]. The density of the potassium formate solution is enough to extract high-density plastics such as polyvinyl chloride (PVC, *ρ* = 1.14–1.56 g/cm^3^) and polyethylene terephthalate (PET, *ρ* = 1.32–1.41 g/cm^3^).

## Method implementation

The method can be used to determine MP abundance in bottom sediments collected by a corer or a grab sampler.

The proposed method involves several main steps:

1. Preliminary wet peroxide oxidation with H_2_O_2_.
2. Filtration through a cascade of three filter funnels (mesh size 333, 174 and 100 µm) to remove fine grained fractions.
3. Extraction of MPs from the sediment sample with potassium formate (density separation).
4. Wet peroxide oxidation with Fenton's reagent and chitin and mineral fraction digestion with HCl.
5. Filtering and drying the sample.
6. Optional density separation.
7. Internal quality control procedures.
8. Detection of MPs with a microscope.
9. Determination of MP abundance in sediment sample.

This method is designed for extracting MPs from lake sediments and quantifying them with a microscope. MPs detected with this method are fibers, films, fragments and beads of 0.174 to 5 mm in size, that passed flotation in potassium formate with density 1.5 g/mL, are resistant to peroxide oxidization, and are positively identified as MPs under a microscope with 40–45x magnification according to Norén [9]. The quality control procedures described in this method involve assessment of the effectiveness of extracting MPs from sediment, determination of the external contamination rate, and evaluation of ways of material loss during extraction and quantification process. The polymer composition of selected MP items should be confirmed by Raman spectroscopy, µFTIR or ATR FTIR analysis to validate results of visual inspection. The method is also suitable for extracting MPs from river and marine sediments.

***Apparatus and Materials***

• 30% Hydrogen peroxide (H_2_O_2_).
• Fenton's reagent: iron (Fe (II)) solution (0.05 M). Dissolve 7.5 g of FeSO_4_•7H_2_O in distilled water into 500 mL volumetric flask, add 3 mL of concentrated sulfuric acid, and make up to the mark with distilled water.
• Potassium formate solution (HCOOK, density 1.5 g/mL). Dissolve 1,155 g of HCOOK in 345 mL distilled water in 2,000 mL beaker. Solution should be vacuum filtered through a double filter system consisting of a filter net with mesh size 174 µm and cellulose slow flow rate filter (pore size 3–5 µm).
• HCl 4.5% solution. Add 63 mL of concentrated HCl in 500 mL volumetric flask and make up to the mark with distilled water.
• Artificial Reference Particles (ARPs). Prepared from a sheet of fluorescent polyethylene terephthalate (PET) bottle with a thickness of around 0.4 mm and the size of the sides around 0.9*0.4 mm.
• Square filter nets of 18*18 cm (mesh size 333, 174 and 100 µm) and 10*10 cm (mesh size 174 µm) are made of a sieve cloth. Round filter nets for blank samples with diameter of 10 cm (mesh size 174 µm) are made of a sieve cloth. Filter nets should be cleaned thoroughly in distilled water, dried, and analyzed under a microscope with 40–45x magnification for the presence of external contamination. Clean filter nets should be stored covered with aluminum foil.
• Filter funnel. Prepared by folding a square filter net made of a sieve cloth (size 18*18 or 10*10 cm) into a cone, inserted into a glass funnel (diameter 10 or 6 cm) and fixed by a wooden clamp.
• A density separator is made of a 150-mm glass funnel with a 50-mm long segment of latex tube at the bottom of the funnel stem and a clamp forceps that is attached to the latex tube to control the flow of liquid from the funnel.

***Supplementary equipment***
Note 1: Materials quantity is presented for analysis of two samples and one blank.
Note 2: All equipment applied should not contain plastic materials where possible. Metal, glass and wood are the most acceptable materials.

• Laboratory balance with accuracy 0.1–0.01 g and measuring range not less than 2000 g.
• Water bath with temperature control.
• 800-mL glass beakers – 7 pcs.
• 2,000 mL glass beaker – 1 pc.
• 500-mL volumetric flask – 2 pcs.
• Metal or ceramic spatula to take an aliquot of chemicals.
• Glass stirring rod with the length of 30–40 cm – 2 pcs.
• 25-mL, 50-mL, 100-mL, and 500-mL volumetric glass cylinders – 1 pc of each.
• Glass funnels 10 cm in diameter – 6 pcs.
• Glass funnels 6 cm in diameter – 4 pcs.
• 25-mL Mohr pipette – 2 pcs.
• Aluminum foil.
• Petrie dish with diameter of 10 cm – 8 pcs.
• Desiccator 30 cm in diameter – 1 pc.
• Cover for desiccator (40 cm × 40 cm) made of a sieve cloth with mesh size 174 µm.
• Ultraviolet lamp for ARPs detection.
• Metal stand rod with cast iron rings and clamps.
• Tweezers – 2 pcs.
• Squirt bottle with distilled water.
• Wood clamp (small clothespin) to fix a filter net on a glass funnel – 10 pcs.

Before the sample analysis, the following information should be recorded in the workbook: date, sample code, blank sample code, and quantity of ARPs to be added into the sample. Other working data should be recorded during the analysis (Supplementary 1).

***Measuring instruments, supplemental equipment for MPs quantification with a microscope***

**Fig. 1:**
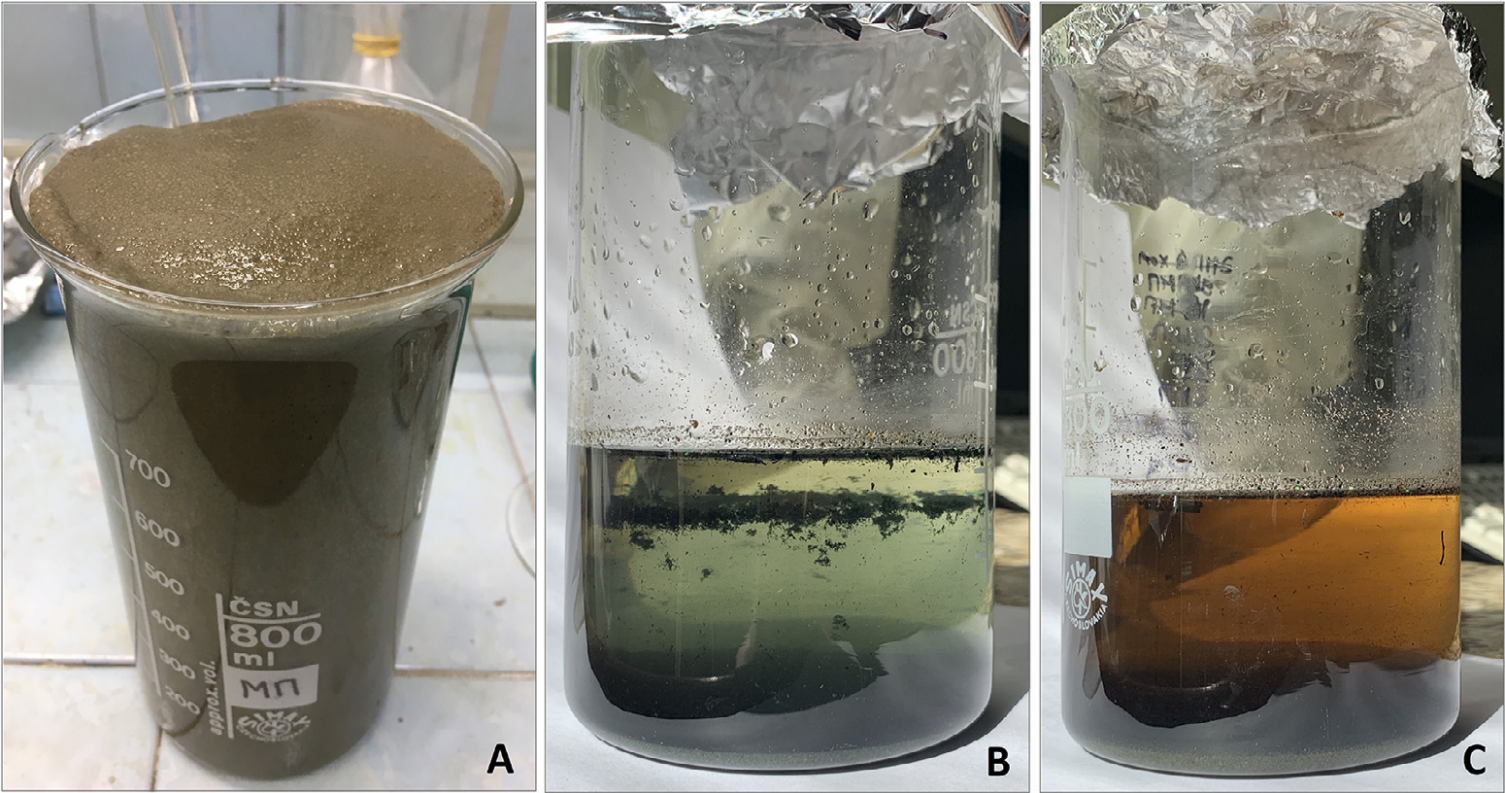
Analysis of sediment sample: (A) preliminary wet peroxide oxidation, (B) and (C) density separation.
  1.4. If the sample overflows the beaker, it is necessary to transfer some part of the sample to another beaker and wait till the end of the reaction. After the reaction is complete, the beaker should be covered with aluminum foil. This step is aimed at disaggregating MPs and sediment particles.
  2. ***Sediment flushing through the filter funnels cascade***
  2.1. Set a blank sample according to section step 7.1.
  2.2. Add 100 mL of distilled water into the beaker containing the sample and mix it with a glass stirring rod. Transfer some part of the sample to the first filter net of the filter funnels cascade consisting of three filter funnels with subsequent decreasing of the mesh size 333, 174, and 100 µm, situated one above the other (Fig. 2).

• Stereomicroscope with magnification from 10× to 40–45×
• Tweezers – 1 pc
• Dental probe (for separating particles from each other) – 1 pc
• Paper clips (for filter net fixation)
• Object plate
• Cover glass
• Cardboard with lined field (1.0*1.0 cm)
• Aluminum foil
• Scotch tape
  1. ***Preliminary wet peroxide oxidization***
  1.1. Mix the wet sediment sample in the sample container with a metal spatula.
  1.3. Weigh 400 g of the wet sediment sample in an 800-mL glass beaker and add 20 ARPs to the sample as an internal standard.
  1.3. Add 50 mL of 30% H_2_O_2_ in small portions (15–25 mL) into the beaker with the sample using a volumetric cylinder. **CAUTION:** oxidation can be violent (Fig. 1(A)), for this reason, it is necessary to control the reaction. The mixture should be stirred with a glass stirring rod to collapse constantly produced bubbles.

**Fig. 2:**
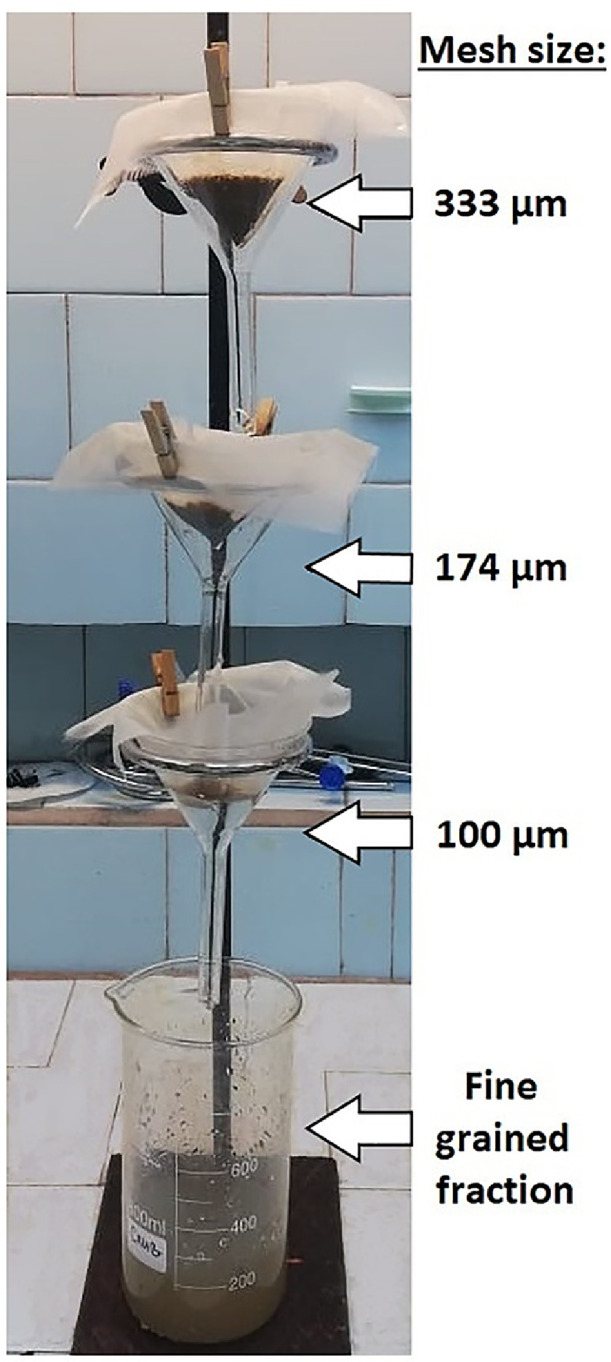
Sediment flushing through the cascade of three filter funnels with subsequent decreasing of the mesh size 333, 174, and 100 µm, situated one above the other.
  2.3. Each filter net should be washed thoroughly with distilled water using the squirt bottle to remove fine grained fraction (mud and silt). The filter nets should be washed thoroughly until only a solid part of the material remains on each filter net. The drain from the last funnel should be redirected into the drain beaker, which should be discarded when it is full.
  2.4. If the filter nets are clogged, transfer the most of the solid material from the filter nets into an 800 mL beaker for further density separation as follows: remove the filter net from the funnel, tilt it over the beaker and scrape the sediment from the filter net into the beaker using a glass stirring rod. Cover the beaker with aluminum foil and set up the filter net back on the funnel.
  2.5. Steps 2.1–2.3 should be repeated until the whole sediment sample is transferred into the beaker. Rinse the beaker that initially contained the sample with distilled water using a squirt bottle and transfer all residuals to the filter funnels cascade. Check the beaker with an ultraviolet lamp to assess possible ARPs loss.
  2.6. Dispense 300 mL of an aqueous potassium formate (*ρ* = 1.5 g/mL) solution into a 500 mL volumetric glass cylinder. Transfer the last sediment portion sediment from the filter nets into the 800 mL glass beaker (see step 2.4). To transfer residuals from the filter nets into the beaker, turn them upside down over the beaker and wash thoroughly with HCOOK solution from the cylinder. Check the filter nets with the ultraviolet lamp to assess the possible ARPs loss. Pour the remaining HCOOK solution from the cylinder into a beaker containing the sample.
  2.7. Filter nets used in section 2 can be reused after the aforementioned preparation procedures (see section Apparatus and materials).
  3. ***Density separation***
  3.1. Mix the HCOOK solution and the sample in the beaker with a glass stirring rod for several minutes to stir up the sediment. Cover the beaker with aluminum foil and let the mixture settle from 2 to 5 days. Under the gravity force, light items (MPs and various organic inclusions) float up and heavier sediment particles settle to the bottom (Fig. 1(B), (C)).
  3.2. Examine the floating items for the ARPs presence and document the ARPs abundance.
    Number the filter nets F1 and F2 and set them to the filter funnels (10*10 cm; mesh size 174 µm) placed one above the other according to the filter net numbers. Discharge the solution into a cascade of two filter funnels carefully (Fig. 3).

**Fig. 3:**
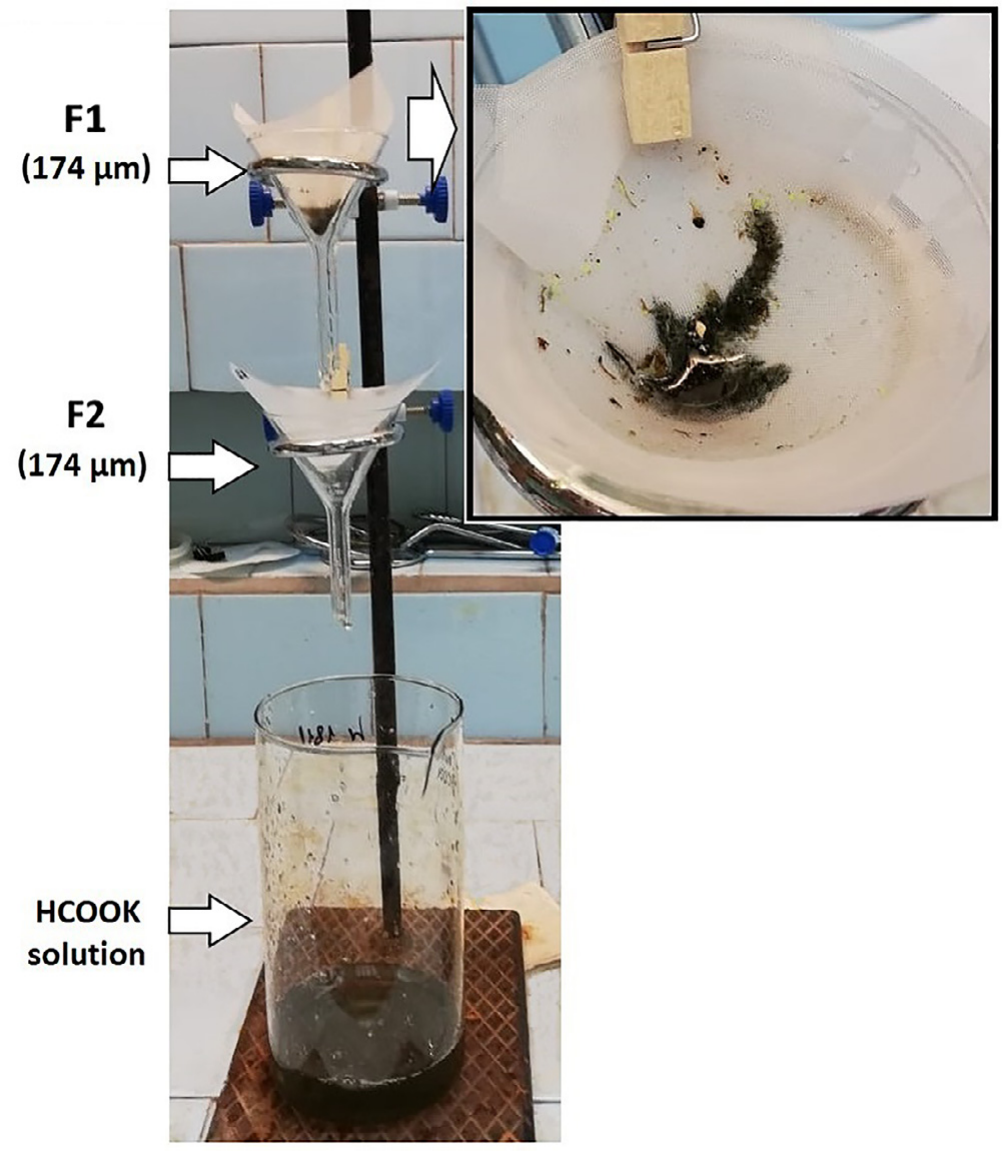
Filtration of HCOOK solution through the cascade of two filter funnels with filter nets F1 and F2 placed one above the other according to their numbers.
  3.3. If less than all the added ARPs are floating, add HCOOK solution utilized in the previous step into the beaker, stir the solution and let the mixture settle for 24 hours. Repeat steps 3.1–3.3 until all added ARPs are detected.
  3.4. Roll the filter nets F1 and F2 into a cone, carefully immerse them in a beaker containing distilled water and wash thoroughly to remove potassium formate.
  3.5. A potassium formate solution can be reused. The solution should be vacuum filtered through a double filter system consisting of a filter net with a mesh size 174 µm and a cellulose slow flow rate filter (pore size 3–5 µm). It can be applied for density separation if its specific density is not lower than 1.5 g/mL.
  4. ***Wet Peroxide Oxidation (WPO)***
  4.1. Place the filter nets F1 and F2 (see step 3.4) containing solids into an 800 mL glass beaker. Add 25 mL of aqueous 0.05 M Fe(II) solution and 25 mL of 30% H_2_O_2_ solution using pipettes into the beaker to digest natural organic matter. **CAUTION**: This mixture is highly reactive. Please, review and follow your laboratory safety practices and policies for handling this mixture before completing this analysis.
  4.2. Cover the beaker containing the mixture with aluminum foil and leave it at room temperature for five minutes before the next step (Fig. 4(B)).

**Fig. 4:**
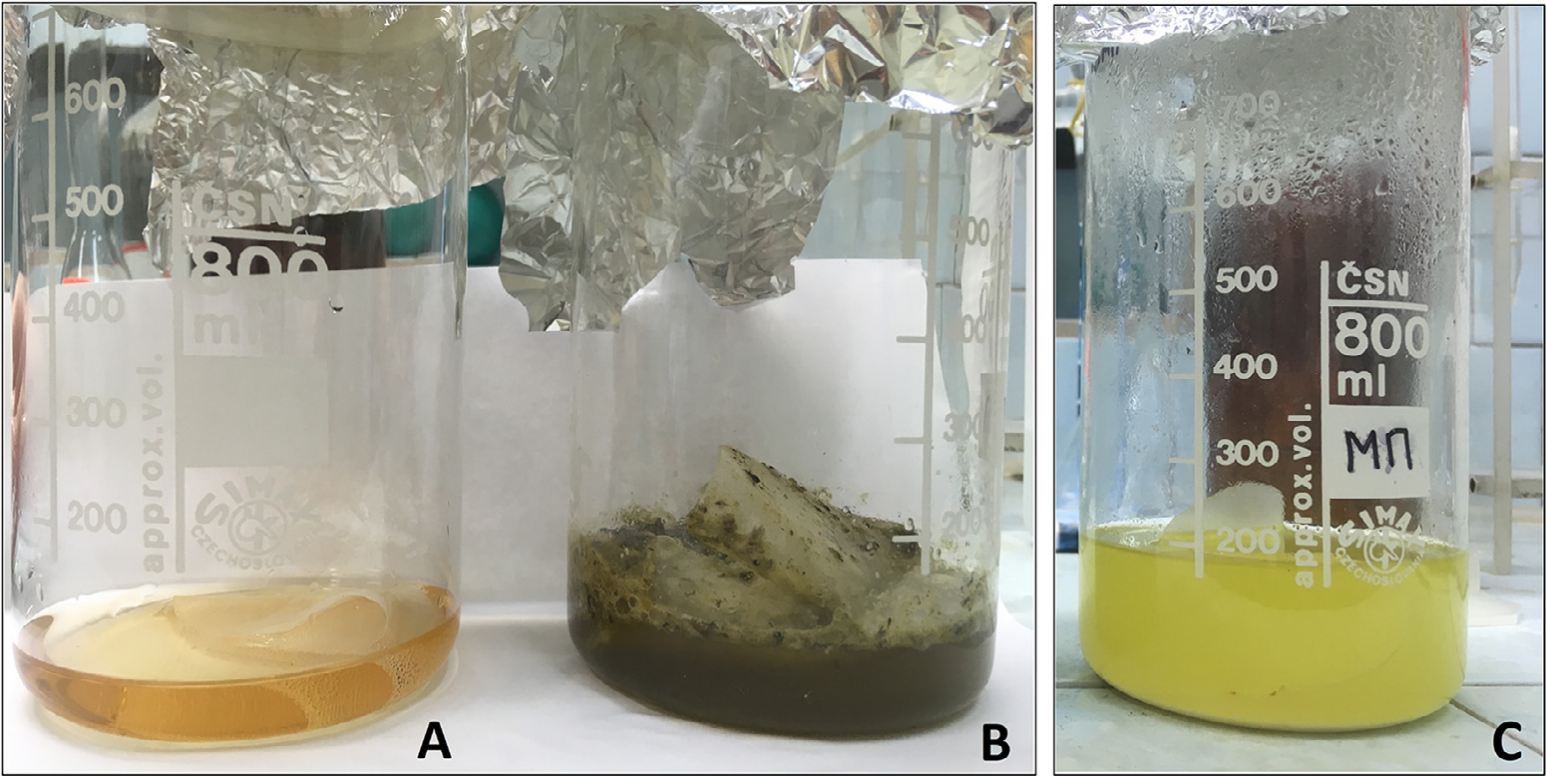
Wet peroxide oxidation: (A) blank sample at the initial stage of oxidation, (B) sample at the initial stage of oxidation, (C) sample after adding HCl solution.
  4.3. Place the beaker with the mixture in a water bath heated to 70 °С. **CAUTION**: This mixture can boil violently. If the mixture is going to overflow the beaker, add several mL of distilled water using a squirt bottle to slow down the reaction.
  4.4. If the natural organic matter is still visible after the reaction is interrupted, add another 25 mL of 30% H_2_O_2_ solution to the beaker with the mixture. Repeat this step until all of the natural organic matter is digested.
  4.5. Let the beaker cool for 2–5 min at room temperature. Add 25 mL of 4.5% HCl solution with a cylinder into the beaker to dissolve the residuary chitin fractions and other mineralized solids (Fig. 4(C)).
  5. ***Filtering and drying the sample***
  5.1. Remove the filter nets F1 and F2 from the beaker using tweezers, wash them thoroughly with distilled water using a squirt bottle, roll into a cone, and dry at room temperature in a desiccator covered with a sieve cloth (Fig. 5). Place the dried filter nets F1 and F2 in a Petri dish for control analysis (mark the Petri dish with the sample code and the remark CONTROL 1).

**Fig. 5:**
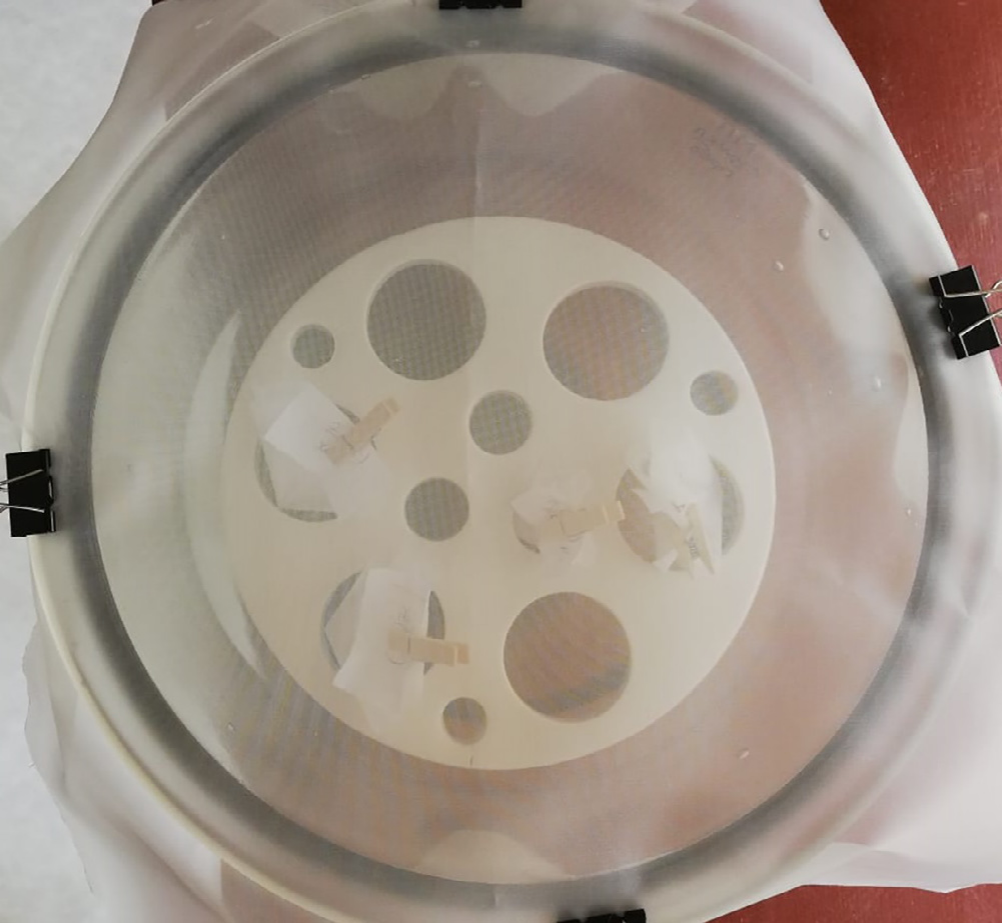
Filter nets drying in a desiccator covered with a sieve cloth.
  5.2. The remaining solution containing MPs should be filtered through a cascade of two filter funnels (10*10 cm; mesh size 174 µm) placed one above the other. Number the filter nets F3 and F4 according to their position on the cascade (Fig. 6).

**Fig. 6:**
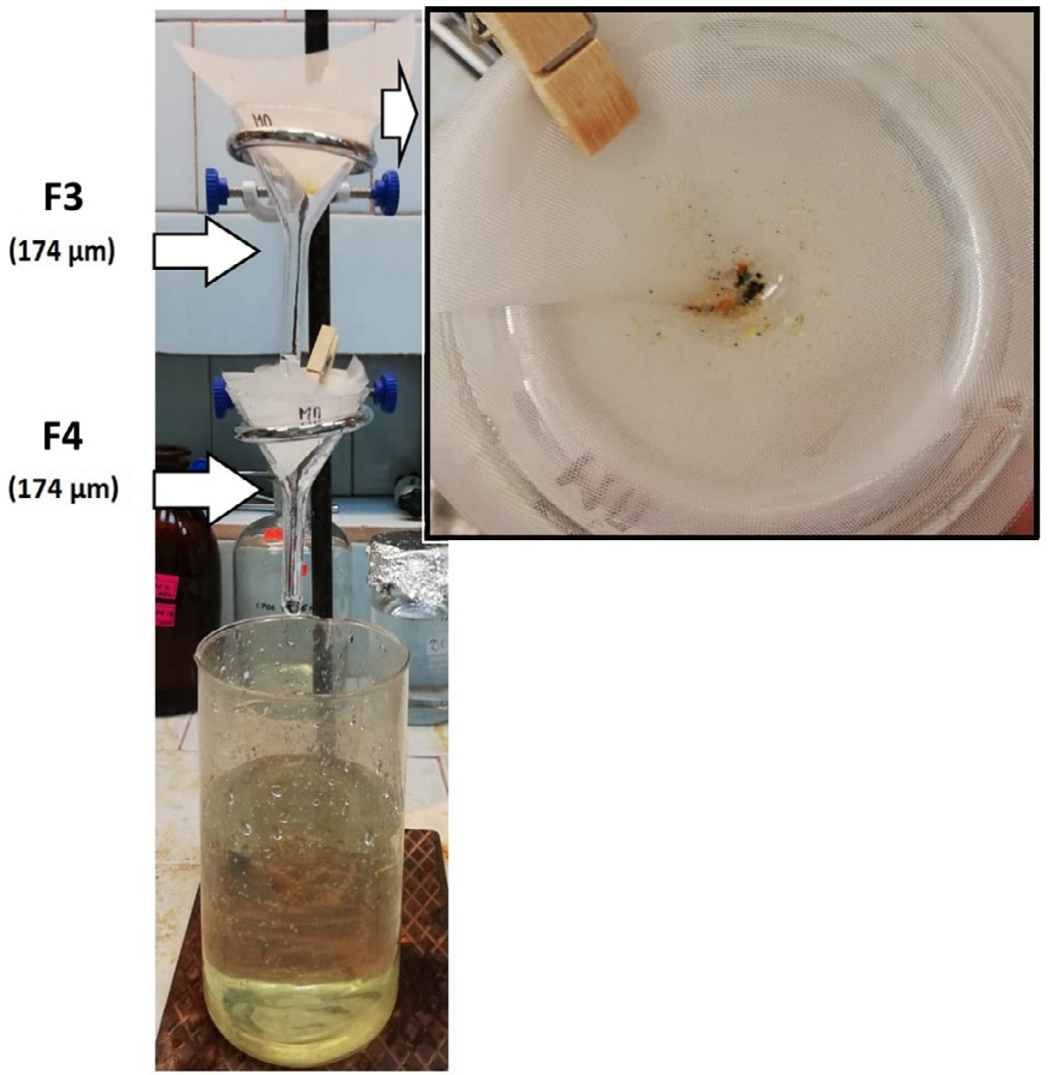
Filtration of the remaining solution through the cascade of two filter funnels with filter nets F3 and F4 placed one above the other according to their numbers.
  5.3. Rinse the beaker multiple times with distilled water to completely transfer all the solids to the filter nets F3 and F4. Check the beaker with the ultraviolet lamp to assess the possible ARPs loss.
  5.4. Rinse the filter nets F3 and F4 with distilled water from the squirt bottle carefully.
  5.5. If optional density separation is not required (there are no sand particles), the filter nets F3 and F4 containing solids should be rolled into a cone and dried at room temperature in a desiccator covered with a sieve cloth (Fig. 5). Place the dried filter nets F3 and F4 in a Petri dish for control analysis (mark the Petri dish with the sample code and the remark PRIMARY).
  6. ***Optional density separation***
  6.1. If a large amount of sand is visible in the sample after oxidation, an optional density separation is required. To transfer all particles from the filter nets F3 and F4 into the density separator (Fig. 7), turn them upside down over the density separator and wash thoroughly with 100 mL HCOOK solution from the cylinder. Cover the density separator with aluminum foil and let the mixture settle for an hour.

**Fig. 7:**
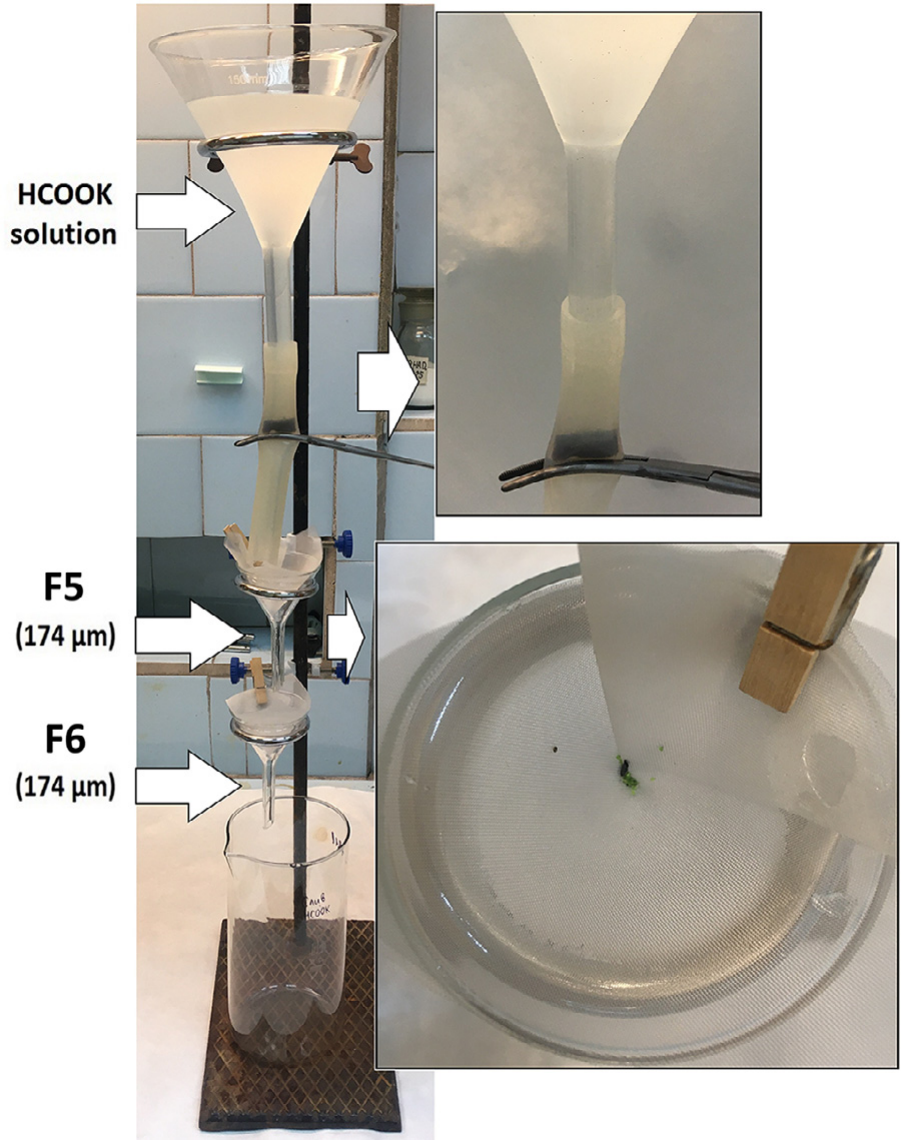
Optional density separation and HCOOK solution filtration through the cascade of two filter funnels with filter nets F5 and F6 placed one above the other according to their numbers.
  6.2. Roll the filter nets F3 and F4 (after step 6.1) into a cone and carefully immerse them into the beaker containing distilled water and wash thoroughly to remove potassium formate. Dry them in a desiccator covered with a sieve cloth at room temperature (Fig. 5). Place dry filter nets in a Petri dish for analysis control (mark the Petri dish with the sample code and the remark CONTROL 2).
  6.3. Release the clamp forceps, pour the settled fractions (Fig. 7) into the beaker and discard.
  6.4. Set the cascade of two filter funnels (10*10 cm; mesh size 174 µm) under the separator and release the clamp forceps to transfer all solids from the surface to the filter nets. Number the filter nets F5 and F6 according to their position in the cascade.
  6.5. Thoroughly wash the density separator with HCOOK solution to transfer all solids to the cascade of two filter funnels (Fig. 7).
  6.6. Roll the filter nets F5 and F6 into a cone carefully and immerse them into the beaker containing distilled water and wash thoroughly to remove potassium formate. Dry them in a desiccator covered with a sieve cloth at room temperature (Fig. 5). Place dry filter nets in a Petri dish (mark the Petri dish with the sample code and the remark PRIMARY).
  7. ***Internal quality control***
  7.1. One blank should be run for every series of five samples. For this purpose, at the beginning of the analysis, a clean round filter net (B1) (mesh size 174 µm) should be placed into a clean Petri dish, moistened in distilled water, kept open throughout the filtration step (Section "Sediment flushing through the filter funnels cascade") and removed after the end of filtration. The blank sample should be marked with the corresponding samples codes and recorded in a workbook (Supplementary 1).
  7.2. The filter net B1 containing the blank sample should be exposed to wet peroxide oxidation and HCl digestion in the same way as the samples (see section "Wet Peroxide Oxidation (WPO)"; Fig. 4(A)). After oxidation, the filter net B1 should be removed from the beaker with tweezers, washed (see step 5.1.), rolled in a cone, and dried at room temperature in a desiccator covered with a sieve cloth (Fig. 5). Place dry filter net in a Petri dish for control analysis. Mark the Petri dish with the sample code and the remark BLANK CONTROL.
  7.3. After oxidation, the remaining solution should be filtered through a cascade of two filter funnels in the same way as it is done for the sample (see section "Filtering and drying the sample"). Number the filter nets B2 and B3, roll in a cone, and dry in a desiccator covered with a sieve cloth at room temperature (Fig. 5). Place dry filter nets in a Petri dish for control analysis. Mark the Petri dish with the sample series code and the remark PRIMARY BLANK.
8. ***Microplastics detection with microscope***
  8.1. Define the following information in the workbook (Supplementary 2): water object, station, sample code, date, and filter net code.
  8.2. Fix the filter net with the sample carefully on the lined cardboard using the paper clips. Use tweezers and a dental probe to separate, move and test items on the filter net surface.
  8.3. Identify MP item according to the recommendations of Norén [9]:
    • Cell structure and other organic forms of items are absent;
    • Fibers should have uniform thickness along the entire length;
    • Items must have a clean and uniform color;
  8.4. Classify MP items into four groups according to their shape: fragments, films, fibers and beads:
    • Fibers – thin elongated items with one dimension significantly greater than the other two (Fig. 8(A));

**Fig. 8:**
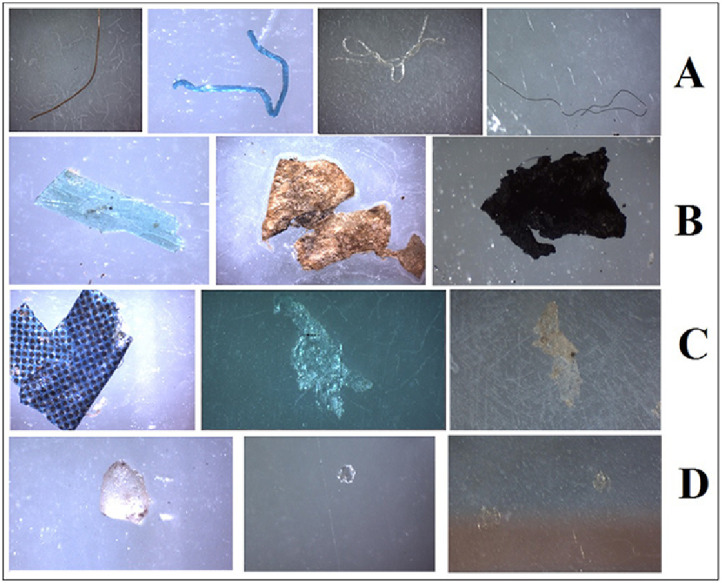
Example of MP types extracted from sediments ((A) fibers, (B) – fragments, (C) films, (D) beads).
    • Fragments – pieces of thick plastics of irregular shape with all three dimensions comparable (Fig. 8(B));
    • Films – sheets of plastic bags and other similar stuff, with their thickness significantly lower than other two dimensions (Fig. 8(C));
    • Beads –three-dimensional items of rounded shape (Fig. 8(D)).
  8.5. Define the MP item color from the thirteen groups: transparent, white, gray, beige, brown, green, yellow, pink, red, blue, goldish, silverish and black. Dark blue, light blue, magenta, purple, cyan are included in the blue category. Raspberry and pink colors are included in the pink category. Orange color is in the red category. Also, include MP items into corresponding category if they have the corresponding color of shining or corresponding transparent color. Note down the MP item color in the workbook (Supplementary 2).
  8.6. Measure the size of MP item (length for the fibers; length/width for all other MPs type) using the built-in microscope scale. Note down the MP item size in the workbook (Supplementary 2). If the largest dimension of the item exceeds 5 mm, it should be identified as mesoplastics except for thin fibers. If the fiber is thinner than 0.1 mm, it should be identified as MPs, regardless of its largest dimension.
  8.7. Note down the quantity of ARPs detected on the filter net F1 in the workbook (Supplementary 2).
  8.8. Prepare MP items for chemical analysis with µFTIR, ATR-FTIR or Raman spectrometry. Make a photo of the selected item. Place a little drop of distilled water (~ *d* = 1.0 mm) on the object plate and transfer the selected MP item to the drop using tweezers. Cover the water drop containing the item with a cover glass, fix it by a scotch tape, pack it in aluminum foil, and sign with the specimen code. Register the specimen in the protocol (Supplementary 3).
  8.9. Repeat steps 8.1–8.8 for all filter nets (F2 and others) of the same sample.
9. ***Determination of microplastic abundance***
  9.1. To determine the MP abundance in a sample, the quantity of items of a certain type and color in the sample ($N^{color,type}$) and the blank ($N_{blank}^{color,type}$) should be calculated. For this purpose, summarize the number of MP items detected on all filter nets by type and color.
  9.2. If optional density separation after peroxide oxidization was not conducted, the number of items of a certain type and color ($N^{color,type}$) in the sample is calculated in the following way:
    $$N^{color,type}=N_{F1}^{color,\;type}\;+N_{F2}^{color,\;type}+N_{F3}^{color,\;type}+N_{F4}^{color,\;type},$$
    where $N_{F1}^{color,\;type}\;$ and $N_{F2}^{color,\;type}$ are the quantities of items of a certain type and color on the “CONTROL 1” filter nets, pcs (see step 3.2. and 5.1.). $N_{F3}^{color,\;type}$ and $N_{F4}^{color,\;type}$ are the quantities of items of a certain type and color on the “PRIMARY” filter nets, pcs (see step 5.5.).
  9.3. If optional density separation step was conducted, calculate the quantity of MP items in the sample according to the formula:
    $$N^{color,type}=N_{F1}^{color,\;type}\;+N_{F2}^{color,\;type}+N_{F3}^{color,\;type}+N_{F4}^{color,\;type}+N_{F5}^{color,\;type}\;+N_{F6}^{color,\;type},$$
    where $N_{F1}^{color,\;type}$ and $N_{F2}^{color,\;type}$ are the quantities of MP items of a certain type and color on the “CONTROL 1” filter nets, pcs (see step 3.2. and 5.1.); $N_{F3}^{color,\;type}$ and $N_{F4}^{color,\;type}$ are the quantities of MP items of a certain type and color on the “CONTROL 2” filter nets, pcs (see step 6.2.); $N_{F5}^{color,\;type}$ and $N_{F6}^{color,\;type}$ are the quantities of MP items of a certain type and color on the “PRIMARY” filter nets, pcs (see step 6.6.).
  9.4. Calculate the quantity of MP items ($N_{blank}^{color,type}$) of a certain type and color in the blank in the following way:
    $$N_{blank}^{color,type}=N_{B1}^{color,type}+N_{B2}^{color,type}+\;N_{B3}^{color,type},$$
    where $N_{B1}^{color,type}$, $N_{B2}^{color,type}$, and $N_{B3}^{color,type}$ are the quantities of MP items of a certain type and color on the corresponding filter nets B1, B2 and B3, pcs (see step 7.2. and 7.3.).
  9.5. Calculate the blank corrected ($N_{BC}^{color,\;\;type}$) quantity of MP items of a certain type and color in the sample as the difference:
    $$N_{BC}^{color,\;\;type}=N^{color,type}-N_{blank}^{color,type}$$
    If the difference is negative, set $N_{BC}^{color,\;\;type}$ to zero.
  9.6. Calculate the quantity of a certain type of MP items (fibers, films, fragments and beads) in the sample ($N^{type}$, pcs) using the formula:
    $$N^{type}=\sum_{color}N_{BC}^{color,\;\;type}$$
  9.7. Calculate the total quantity of MP items in the sample ($N^{sample}$, pcs) using the formula:
    $$N^{sample}=\sum_{type}N^{type}$$
  9.8. Calculate the MP abundance (A) in sediment sample as the quantity of MP items per kilogram of dry sediment weight (pcs/kg DW):
    $$A\;=\;\frac{N^{sample}\cdot 100\;\%}{m^{sample}\left(100-W\right)},$$
    where $N^{sample}$ is the quantity of MP items of all types and colors in the sample, pcs.; $m^{sample}$ is the sample weight, kg; W is the sample wetness, %.

## Quality assurance and contamination control

Quality control procedures are important during the analysis of MPs in environmental samples. Overestimation (false positives) can occur as a result of background contamination of a sample with MPs from the indoor air, while underestimation emerges as a losing of analytical material during analysis. Thus, internal quality control is essential for accurate identification of MPs quantities throughout the sample processing. In order to assess background contamination, one blank should be run for every five samples. Possible losing of analytical material during analysis can be controlled by ARPs quantification during different steps of analysis.

*Background contamination.* To reduce false positives in the results, it is essential to eliminate potential sources of background contamination where possible. The following steps should be performed in this regard:

1. The whole process of sample handling should be conducted in a clean room conditions with controlled air circulation, or alternatively (if it is not possible to use a clean room) using a laminar flow workbench.
2. All filter nets used for MPs analysis should be cleaned thoroughly in distilled water, dried, and analyzed under a microscope with a 40–45x magnification for the presence of external contamination. Clean filter nets should be stored covered with aluminum foil.
3. All equipment and laboratory ware used for sampling and analysis of MPs should not contain plastic materials where possible. Unpainted metal, glass and wood are the most acceptable materials. Laboratory ware should be thoroughly washed with distilled water, dried, and stored covered with aluminum foil before the analysis.
4. It is preferable to use a 100% cotton lab coat instead of synthetic textiles during sampling or sample processing.
5. It is necessary to clean the surfaces in the lab room as often as possible.

Sometimes, the analytical material can be lost due to the retention of MP items on the surface of laboratory ware and filter nets during the analysis of MPs. Therefore, it is necessary to carefully rinse laboratory ware with distilled water and thoroughly examine it in order to detect possible retention of MPs. The MPs can be retained in the Petri dish where the sample was stored, therefore the Petri dish should be occasionally analyzed under a microscope with 40–45x magnification.

*Impact of reagents.* A number of studies have confirmed that some reagents applied to digest organic matter can damage or decompose some kinds of polymers under particular conditions [3,6] resulting in underestimation of the MPs content. Digestion of organic matter using the Fenton's reagent applied in this method is an exothermic reaction producing temperatures up to 89 °C [8] which can have an effect on different types of polymer materials. It has been indicated that polyamide (PA), polypropylene (PP), and polycarbonate (PC) particles have some visible changes (discoloration, shrinkage, partial digestion) in 30% H_2_O_2_ at 70 °C [3,^6^,10]. For example, a 6.2% loss in size was found for polyethylene (PE) and PP particles <1 mm in size [10] as well as a color change of PET [4,10]. PA particles are nonresistant to such reagents and can be destroyed in 30% H_2_O_2_ solution [3,6]. Furthermore, it has been found that almost all commonly used plastic types (PE, PP, PET, PVC and polystyrene) are resistant to 5% HCl solution [4,6] except PA which is destroyed even in diluted hydrochloric acid [6]. As a result, PA content can be underestimated when using the proposed method. Such polymer types as PE, PP, PC and PET can undergo shrinkage, deformation and discoloration during analysis.

*Internal quality control.* Internal quality control is essential procedure for finding and eliminating ways of losing analytical material during the analysis of MPs. The proposed method includes internal control procedures aimed at controlling the quality of analysis using ARP-particles. The quality analysis with ARPs allows to estimate and control the following parameters and procedures: effectiveness of MPs extraction from the sample; the quality of MPs transfer during analysis; the cross-contamination rate between the samples; and tracking losses at the various stages of analysis.

The effectiveness of MPs extraction from the sediment is estimated by counting the quantity of ARP particles after the extraction step. The rate of ARPs extracted to ARPs added and normalized by 100% indicates the rate of extraction. If the rate is lower than 100%, this indicates the retention of ARPs and MPs by the sediment. If the result is 95% or higher, this suggests a good extraction effectiveness. A rate lower than 95% indicates some problems with extraction and sequential extraction is recommended.

The quality of MPs transfer during analysis can be controlled at each stage. However, the obligatory stages are performed immediately after the extraction step and during the analysis of the sample under the microscope. If less than all ARP-particles were recovered, this indicates that some portion of MP in the sample was also lost during analysis.

The losses should be interpreted in the following ways:

• ≤ 5%, Low loses. The obtained results are of good quality and don't require correction.
• 5–15%, Moderate loses. The MPs abundance in the sample seems to be underestimated. It is necessary to divide the result of the abundance of MP for each of the forms by the rate of the extracted ARPs to the added ARPs, or indicate that the results may be underestimated.
• > 15%, High loses. It indicates that an analytical mistake occurred. In this case, tracking of losses at various stages of analysis should be conducted or sequential extraction procedures should be applied.

Cross contamination can occur during sample processing in case of low quality of laboratory ware preparation between samples. This can be recognized if the number of ARPs found exceeds the ARPs added. In this case, the quality of laboratory ware preparation should be critically examined and the adjacent samples re-analyzed.

To track loses at various stages of analysis, all laboratory ware used, as well as the Petri dish in which the sample was kept, should be thoroughly examined using an ultraviolet lamp. If ARP-particles have been found on laboratory ware, the operator should take more care when transferring the material during analysis, and this ware should be washed-out repetitively.

This method was statistically tested and the following results were obtained. After the extraction step, extraction effectiveness was 98.0 ± 2 % (*р* = 0.05; *n* = 16), indicating the perfect extraction rate with potassium formate. However, during analysis under the microscope, the quantity of ARPs decreased to 92.0 ± 4% (*р* = 0.05; *n* = 20), indicating the loss of a certain quantity of the material at the second stage of determination. Thus, the values presented can be underestimated by around 6 %.

## Method validation

A sequential MPs density separation from the sediment was performed to assess the extraction effectiveness with potassium formate. The sediment samples used in the experiment were collected in Lake Onego and differ from each other in the sediment type and the organic matter content (Table 1). Organic carbon (OC) content varied between 0.3 and 20.5%, with the maximum concentration in sample K3, due to the anthropogenic impact of the pulp and paper mill at this station.

**Table 1:** Specifications of the sediment samples.

Samples	OC, %	Sediment type
S5	0.3	Medium sand
W2	2.6	Silt with sand
GU	3.1	Silt
K3	20.5	Silt with sand

MPs from each sample were extracted with the proposed method. Density separation (see section "Density separation") was conducted three times in order to estimate the quantity of MP items remaining after each extraction. In this regard, a filtered potassium formate solution was added back into the beaker containing the sediment repeatedly after the previous density separation. The filter nets with solids were oxidized, filtrated and dried after each step of the experiment (see sections "Wet Peroxide Oxidation (WPO)" and "Filtering and drying the sample"). MPs on each filter net were counted and identified with the microscope (see sections "Microplastics detection with microscope" and "Determination of microplastic abundance"

**Fig. 9:**
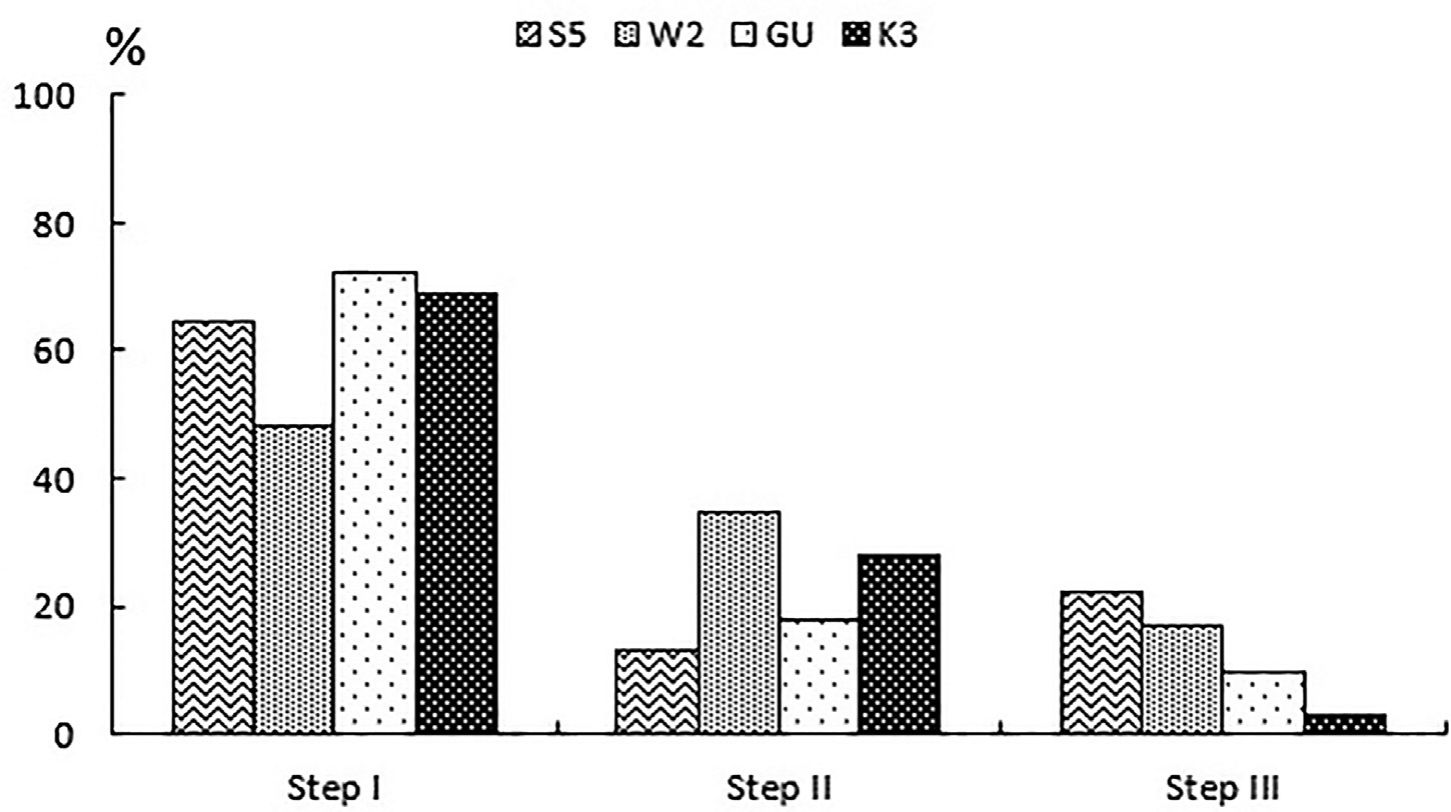
Percentage of MPs extracted at each step of the experiment.

The percentage of MPs extracted at each step were calculated assuming all three steps represent 100% (Fig. 9).

At the first step, the majority of MP items were extracted (64±11% (CI, n=4, p=0.05)) regardless of the sediment grain size and organic carbon content. At the second and third steps, the quantity was consistently lower and accounted to 23±10% (CI, n=4, p=0.05) and 13±9% (CI, n=4, p=0.05), respectively. Thus, three steps of sequential extraction seem to be a good way for complete extraction of MPs from sediments. However, in order to optimize the quantity of extraction steps depending on the sediment retention ability to MPs, they can be controlled by the quantity of ARPs extracted at each step (see step 3.3 and "Quality assurance and contamination control" section for details).

## Declaration of Competing Interest

The authors declare that they have no known competing financial interests or personal relationships that could have appeared to influence the work reported in this paper.

## Appendix Supplementary materials

Image, application 1
