# Supplementary material for: Method for microplastics extraction from Lake sediments
Source: MethodsX. 2020 Nov 12;7:101140. doi: 10.1016/j.mex.2020.101140 (PMC7691543; doi:10.1016/j.mex.2020.101140)
Supplement: Supplementary file 1 [file mmc1.docx]

**Supplementary**

**Supplementary 1.** Workbook for documenting the microplastics separation from bottom sediments

For each analyzed sample the following information should be noted:

- Filtration date
- Sample code
- Blank sample series code
- Quantity of added and found ARPs
- Settling time with density separation (day)
- Oxidation date
- Quantity of filter nets for control test and sample: control and primary

**Workbook example:**

Filtration date: 30.09.2019

Sample code: **Z4**, series 1 (S1), blank sample code: **S1_blank_**

Quantity of added ARPs: 20; quantity ARPs found after density separation: 20

Settling time with density separation (day): 3

Oxidation date: 03.10.2019

Quantity of blank sample filter nets: BLANK control1 (B1), BLANK PRIMARY (B2 and B3). **Total:** 3

If an optional density separation after peroxide oxidization was not conducted, the record should be follows:

Quantity of sample filter nets: control1 (F1 and F2), PRIMARY (F3 and F4). **Total:** 4

If an optional density separation was performed, the record should be follows:

Quantity of sample filter nets: control 1 (F1 and F2), control 2 (F3 and F4), PRIMARY (F5 and F6). **Total:** 6

**Supplementary 2.** Workbook template for microplastic quantification with microscope

| Object, station | Petrozavodsk bay, station Р5 | | | | | | | | |
| --- | --- | --- | --- | --- | --- | --- | --- | --- | --- |
| Date | 30.08.2018 | | | | | | | | |
| Sample type | Bottom sediments | | | | | | | | |
| № particle | Bead | Fragment | Film | Fiber | Color | Size, mm | Chemical analysis  (Raman spectrometry) | Specimen code | ARP^1^, pcs. |
| 1 |  |  |  | + | blue | 0.2 | + | 1mp_1 | 20 |
| 2 | + |  |  |  | white | 0.2*0.2 |  |  |  |
| 3 |  |  | + |  | transparent | 0.5*0.3 | + | 1mp_2 |  |
| 4 |  |  |  | + | red | 0.6 |  |  |  |
| 5 |  |  |  | + | blue | 0.6 |  |  |  |
| 6 |  |  |  | + | blue | 0.5 |  |  |  |
| Total |  | | | | | | | | |

^1^ – control

**Supplementary 3**. Protocol template for chemical analysis of the sample.

| **Sample type** | Bottom sediments | | |
| --- | --- | --- | --- |
| **Sample** | Station Р5 | | |
| **№** | **Specimen code** | **Particle characteristic** | **Analysis results** |
| 1 | 1mp_1 | blue fiber 4.0 mm |  |
| 2 | 1mp_2 | transparent fiber from a tangle 10.0 mm |  |
| 3 | 1mp_3 | blue fiber 1.25 mm |  |
| 4 | 1mp_4 | green fragment 1.5*0.5 mm |  |
| 5 | 1mp_5 | blue film 1.0 mm |  |
| 6 | 1mp_6 | red fiber 16.0 mm |  |
| 8 | 1mp_7 | white fiber 3.0 mm |  |
| 9 | 1mp_8 | transparent fragment 2.0*1.0 mm |  |
| 10 | 1mp_9 | green fragment 0.25*0.25 mm |  |
| 12 | 1mp_10 | silver bead 0.3*0.3 mm |  |
| 13 | 1mp_11 | transparent film 1.2*0.5 mm |  |
